# Exploring the association between circadian rhythms and osteoporosis: new diagnostic and therapeutic targets identified via machine learning

**DOI:** 10.3389/fmolb.2025.1614221

**Published:** 2025-06-26

**Authors:** Jian Du, Tian Zhou, Ran Meng, Wei Zhang, Jin Zhou, Wei Peng

**Affiliations:** ^1^ Senior Department of Orthopedics, The Fourth Medical Center of PLA General Hospital, Beijing, China; ^2^ Graduate School, Hebei North University, Zhangjiakou, China; ^3^ Senior Department of Health Service, The Eighth Medical Center of PLA General Hospital, Beijing, China

**Keywords:** osteoporosis, circadian rhythm, machine learning, key genes, diagnostic model

## Abstract

**Background:**

Osteoporosis (OP) is a systemic metabolic bone disease that may increase the risk of disability or death. Increasing evidence suggests that circadian rhythms play an important role in OP, yet the specific mechanisms remain unclear. Therefore, this study aims to utilize bioinformatics and machine learning algorithms to identify novel diagnostic biomarkers related to the circadian rhythm in OP, providing new targets for early diagnosis and treatment of OP.

**Methods:**

The OP dataset GSE56815 was downloaded from the GEO database, differential expression analysis was performed to identify differentially expressed genes (DEGs) between OP and control samples. DEGs were intersected with circadian rhythm-related genes (CRRGs) to obtain circadian rhythm-related differentially expressed genes (CRRDEGs), which were subjected to Gene Ontology (GO) and Kyoto Encyclopedia of Genes and Genomes (KEGG) enrichment analyses. Four machine learning algorithms were applied to identify key genes for constructing a diagnostic model. The diagnostic performance of the model was validated by plotting receiver operating characteristic (ROC) curves using the GSE7158 dataset. Gene set enrichment analysis (GSEA) was performed on the key genes. Single-sample gene set enrichment analysis (ssGSEA) was used to analyze immune cell infiltration and explore the correlation between key genes and immune cells. Drug-gene interaction networks and competitive endogenous RNA (ceRNA) networks were constructed using the key genes.

**Results:**

A total of 140 CRRDEGs were identified. By comparing four machine learning algorithms, the top five genes from the SVM algorithm (ECE1, FLT3, APPL1, RAB5C and FCGR2A) were determined as key genes for OP. The diagnostic model based on these five key genes demonstrated high diagnostic performance, with AUC of 0.904 for the training set and 0.887 for the validation set. Immune cell infiltration analysis revealed that Type 2 T helper cells and CD56dim natural killer cells were significantly upregulated in the OP group, while activated dendritic cells were significantly downregulated. The drug-gene interaction network and ceRNA network constructed based on the key genes revealed potential therapeutic targets for OP.

**Conclusion:**

This study identified ECE1, FLT3, APPL1, RAB5C and FCGR2A as circadian rhythm-related novel diagnostic biomarkers for OP, providing new insights for further understanding the early diagnosis and treatment of OP.

## 1 Introduction

Osteoporosis (OP) is a common systemic metabolic bone disease characterized by decreased bone mass and microstructural damage, leading to an increased risk of bone fragility and fractures (Letarouilly et al., 2019; Barbuto et al., 2024). Clinically, OP-related fractures are one of the leading causes of disability and death in elderly patients (Ensrud and Crandall, 2024; Stromsnes et al., 2024). With the continuous growth of the global population, the prevalence of age-related chronic diseases is gradually increasing (Hu et al., 2025). Epidemiological surveys indicate that the prevalence of OP in individuals over 50 years old is 19%, and in those over 65 years old, it is 32% (Shen et al., 2022). Due to the asymptomatic nature of early-stage OP, it is often diagnosed only when fractures occur, imposing significant health and economic burdens on patients (Qi et al., 2025). Therefore, early detection, prevention, and diagnosis of OP have become crucial public health issues.

Circadian rhythms are endogenous regulators present in both the central nervous system and peripheral tissues, with a cycle of approximately 24 h (Liu et al., 2024; Brécier et al., 2023). In the body, circadian rhythms influence various biological and physiological processes, such as sleep, metabolism, blood pressure, heart rate, cell cycle, and bone tissue growth (Stothard et al., 2020; Tian and Ming, 2022). Increasing research indicates that circadian rhythms play an important role in bone remodeling and growth (Mei et al., 2024; Kikyo, 2024). For example, circadian rhythms can affect the concentration of bone turnover markers (BTMs) in plasma, thereby influencing the bone remodeling process (Redmond et al., 2016). The circadian rhythm gene BMAL1 has been shown to inhibit osteoclast formation by suppressing the NF-κB signaling pathway, thus affecting osteogenesis ability (Li et al., 2018). Additionally, the circadian rhythm gene REV-ERB can impact the proliferation and differentiation of bone marrow mesenchymal stem cells (BMSCs) into osteoblasts, and its agonists have the ability to inhibit osteoclast formation and bone loss (Song et al., 2018). However, the mechanisms and roles of circadian rhythms in OP remain unclear. Therefore, studying the effect of circadian rhythms on OP is crucial for further understanding the pathogenesis of OP.

**FIGURE 1:**
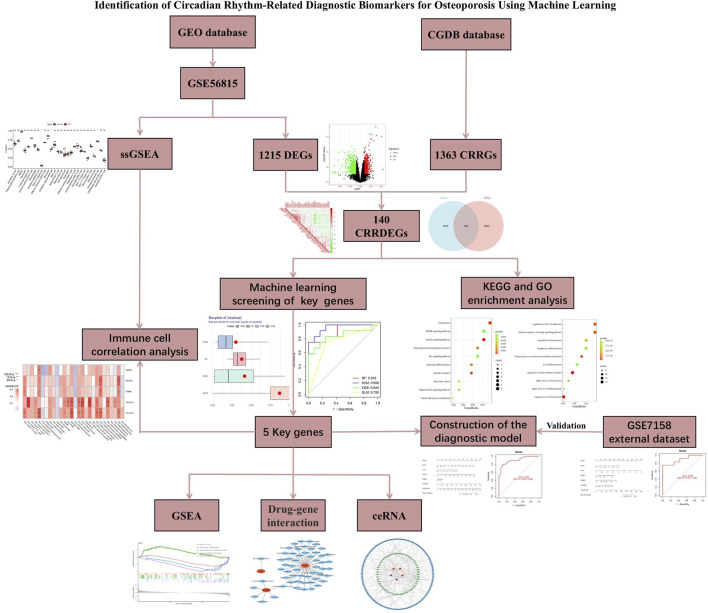
The flowchart of this study.

Machine learning has been widely applied in the medical field, particularly in disease prediction, drug target discovery, and personalized diagnostics (Xuan et al., 2023; Weintraub et al., 2018). However, its application in the diagnosis and treatment of OP remains limited. In this study, we aimed to identify circadian rhythm-related biomarkers for OP using bioinformatics and machine learning techniques (Figure 1). We first obtained OP datasets from the GEO database and intersected it with circadian rhythm-related genes (CRRGs) to identify circadian rhythm-related differentially expressed genes (CRRDEGs). Then, four machine learning algorithms were applied to identify key genes and construct a diagnostic model for OP. Meanwhile, the diagnostic value of the model and key genes was validated using the training set. Furthermore, immune cell infiltration analysis was performed using single-sample gene set enrichment analysis (ssGSEA), and the correlation between key genes and immune cells was explored. Finally, a drug-gene interaction network and competitive endogenous RNA (ceRNA) network were constructed using the key genes. These results may offer novel insights into potential strategies for the early diagnosis and treatment of OP.

## 2 Materials and methods

### 2.1 Data collection

In this study, the datasets GSE56815 and GSE7158 were obtained from the GEO database (https://www.ncbi.nlm.nih.gov/geo/). GSE56815 was used as the training set and included peripheral blood mononuclear cell (PBMC) samples from 40 patients diagnosed with OP and 40 healthy controls. GSE7158 served as the validation set and contained PBMC samples from 12 OP patients and 14 healthy controls. Additionally, 1,363 CRRGs were collected from the CGDB database (http://cgdb.biocuckoo.org).

### 2.2 Circadian rhythm-related differentially expressed genes

Differential expression analysis was performed using the “limma” package in R to identify differentially expressed genes (DEGs) between OP samples and control samples. The filtering criteria were |log FC| >0.1 and *P* < 0.05. The results were then visualized using the “pheatmap” and “ggplot2″ packages in R. Additionally, the intersection of DEGs and CRRGs was obtained to identify CRRDEGs, which were displayed using a Venn diagram.

To evaluate whether the overlap between DEGs and circadian genes was statistically significant, we performed Fisher’s exact test using the total gene set. The test assessed the enrichment of CRRGs within the DEGs.

### 2.3 Functional enrichment analysis

Gene ontology (GO) enrichment analysis and Kyoto Encyclopedia of Genes and Genomes (KEGG) pathway enrichment analysis were performed on CRRDEGs using the “clusterProfiler” package in R. The filtering criterion was adjusted *P* < 0.05 to explore the biological functions of the CRRDEGs.

### 2.4 Machine Learning for Key Gene Selection

In this study, four machine learning algorithms were used to select key genes from the circadian rhythm-related differentially expressed genes (CRRDEGs): Support Vector Machine (SVM), Random Forest (RF), Generalized Linear Model (GLM) and Extreme Gradient Boosting (XGB). Specifically, the “caret” package in R was used to build the models, while the “DALEX” package in R was used to generate residual distribution results for model interpretation. Additionally, the “pROC” package in R was employed to plot receiver operating characteristic (ROC) curves to evaluate the accuracy of the prediction models. Finally, by determining the best-performing model, the top five most important genes were selected as key genes.

### 2.5 Construction and validation of the diagnostic model

A disease diagnostic model was constructed using the key genes. Firstly, the “rms” package in R was used to build a nomogram for the diagnostic model, and the calibration curve was employed to verify the accuracy of the model. Next, Decision Curve Analysis (DCA) was used to assess the clinical utility of the model. Additionally, the Receiver Operating Characteristic (ROC) curve was applied to predict the diagnostic value of the key genes in OP. Finally, the diagnostic performance of the model was evaluated using the training set GSE7158. This comprehensive analysis ensures the models effectiveness in distinguishing OP from control samples and its potential for clinical application.

### 2.6 Gene set enrichment analysis of the key genes

To further explore the potential regulatory pathways of the key genes in OP, GSEA analysis was performed using the “GSEA” package in R, with *P* < 0.05 considered as significantly enriched.

### 2.7 Immune cell infiltration analysis

Immune cell infiltration analysis was performed using ssGSEA. Additionally, the “GSVA” package in R was used to study the relationship between key genes and immune cells, and the results were visualized using the “ggplot2″ package in R.

### 2.8 Construction of the drug regulation network and ceRNA network

The drug-gene interactions between the key genes and potential drugs were analyzed using the Drug-Gene Interaction Database (DGIdb) (https://www.dgidb.org/), and the data results were output. Then, drugs labeled as ‘Approved’ under ‘regulatory approval’ were imported into Cytoscape software for visualization. Additionally, miRNAs associated with the key genes were predicted using the miRanda (http://www.microrna.org/), miRDB (http://mirdb.org/), and TargetScan (http://www.targetscan.org/) databases. Subsequently, lncRNAs related to the key genes were predicted using the spongeScans database (http://mirtoolsgallery.tech/mirtoolsgallery/node/1798). Finally, the ceRNA network was constructed using Cytoscape software, and the results were visualized.

## 3 Results

### 3.1 Identification of CRDEGs

**FIGURE 2:**
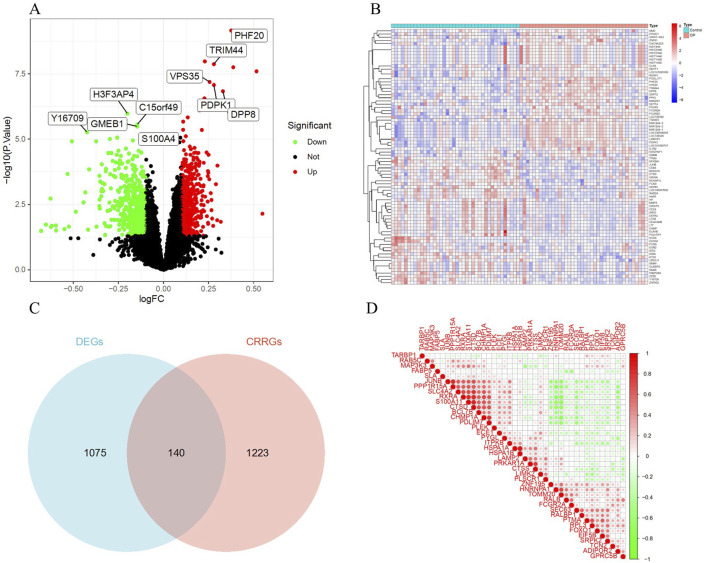
Identification of DEGs. **(A)** Volcano plot of DEGs. Red indicates upregulated genes, green indicates downregulated genes, and black indicates non-significant genes. **(B)** Heatmap of the top 40 DEGs. Red indicates upregulated genes, and green indicates downregulated genes. **(C)** Venn diagram of the intersection of DEGs and CRRGs. **(D)** Gene correlation heatmap of the top 40 CRRDEGs.

A total of 1,215 DEGs were identified from the OP and control group samples, with 579 upregulated genes and 636 downregulated genes (Supplementary Table S1). A volcano plot (Figure 2A) was used to visualize the DEGs. A heatmap was created to visually display the top 20 upregulated and 20 downregulated genes (Figure 2B). Additionally, the intersection of DEGs and CRRGs revealed 140 CRRDEGs (Supplementary Table S2), with 71 upregulated and 69 downregulated genes (Figure 2C). A gene correlation heatmap was used to present the top 40 CRRDEGs (Figure 2D).

To assess the statistical significance of this overlap, Fisher’s exact test was performed using the full set of 14,208 profiled genes. The test yielded a p-value of 0.0191, suggesting a non-random enrichment of CRRGs among DEGs.

### 3.2 Functional enrichment analysis

**FIGURE 3:**
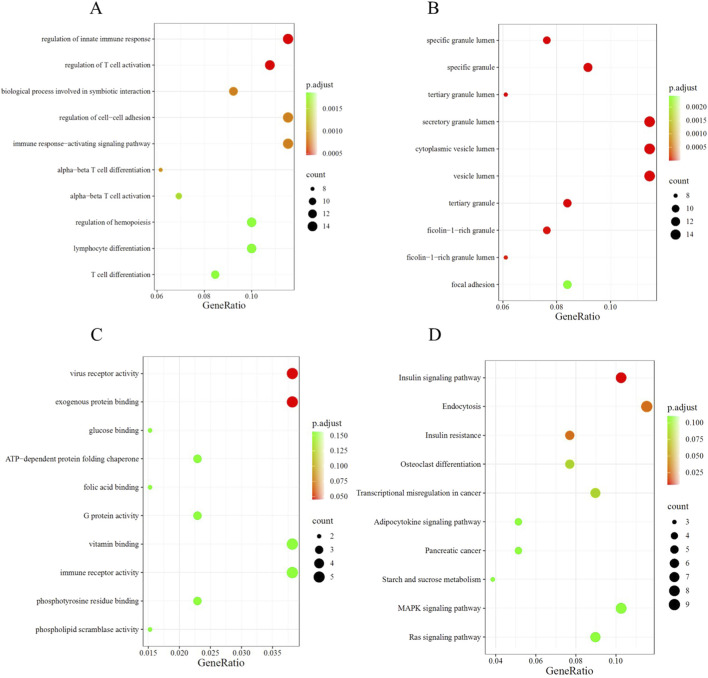
Functional enrichment analysis. **(A)** Bubble plot of BP enrichment analysis. **(B)** Bubble plot of CC enrichment analysis. **(C)** Bubble plot of MF enrichment analysis. **(D)** Bubble plot of KEGG enrichment analysis.

The biological significance of CRRDEGs was further analyzed through GO and KEGG enrichment analyses. GO enrichment analysis revealed that, in biological processes (BP), CRRDEGs were mainly enriched in processes such as regulation of innate immune response, regulation of T cell activation, and biological process involved in symbiotic interaction (Figure 3A). In cellular components (CC), CRRDEGs were mainly enriched in processes such as specific granule lumen, specific granule and tertiary granule lumen (Figure 3B). In molecular functions (MF), CRRDEGs were mainly enriched in processes such as virus receptor activity and exogenous protein binding (Figure 3C). Furthermore, KEGG enrichment analysis indicated that CRRDEGs were primarily enriched in signaling pathways such as Insulin signaling pathway, Endocytosis and Insulin resistance (Figure 3D) (Supplementary Table S3).

### 3.3 Machine Learning for Key Gene Selection

**FIGURE 4:**
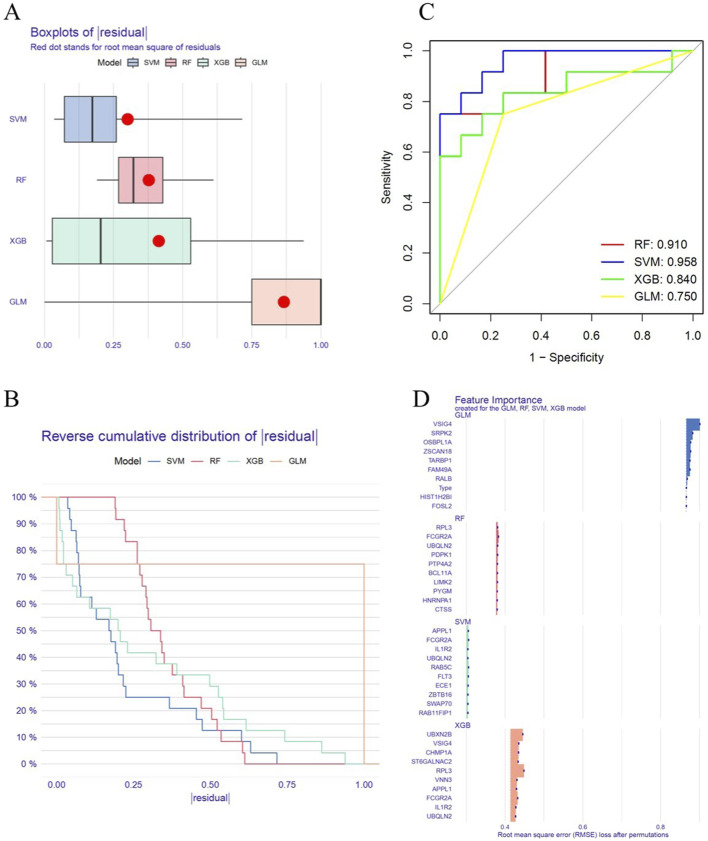
Machine Learning for Key Gene Selection. **(A)** Residual plot for the RF, SVM, GLM and XGB models. **(B)** Residual cumulative distribution plot for the RF, SVM, GLM and XGB models. **(C)** ROC curve analysis for the RF, SVM, GLM and XGB models. **(D)** Feature importance generated by the RF, SVM, GLM and XGB models.

In this study, four machine learning algorithms (RF, SVM, GLM and XGB) were employed to identify key genes related to circadian rhythm in OP. Residual analysis showed that the SVM model had the lowest residual values, while the GLM model had the highest (Figures 4A,B). The ROC curve indicated that the SVM model achieved the highest AUC value (0.958), outperforming RF (0.910), XGB (0.840), and GLM (0.750), suggesting superior classification performance (Figure 4C). In addition, we compared the feature importance profiles across all models (Figure 4D).

To reduce the risk of overfitting, five-fold cross-validation was applied and hyperparameter optimization was performed during model training. Based on the above analysis, the SVM model was identified as the optimal algorithm. Subsequently, the top five genes ranked by the SVM model (ECE1, FLT3, APPL1, RAB5C and FCGR2A) were selected as key genes (Supplementary Table S4) and used to construct a diagnostic model for OP.

### 3.4 Construction and validation of the diagnostic model

**FIGURE 5:**
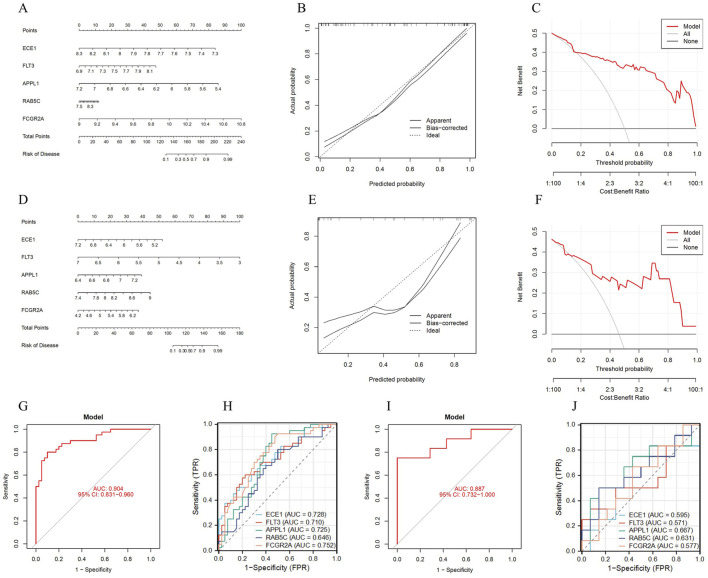
Construction and validation of the diagnostic model. **(A)** Nomogram of the diagnostic model. **(B)** Calibration curve of the diagnostic model. **(C)** DCA curve of the diagnostic model. **(D)** Nomogram of the diagnostic model for the validation set. **(E)** Calibration curve of the diagnostic model for the validation set. **(F)** DCA curve of the diagnostic model for the validation set. **(G)** ROC curve analysis of the diagnostic model. **(H)** ROC curve analysis of the key genes. **(I)** ROC curve analysis of the diagnostic model for the validation set. **(J)** ROC curve analysis of the key genes for the validation set.

To further improve the clinical applicability of diagnosing OP, a nomogram diagnostic model was constructed using the five key genes (Figure 5A). The calibration curve showed that the calibration dashed line was nearly overlapping with the model’s diagonal line, indicating a high predictive value (Figure 5B). DCA results indicated that the model curve was higher than both the ALL and None curves, suggesting high clinical utility (Figure 5C). Furthermore, the models AUC value was 0.904 (95% CI: 0.831–0.960), indicating high diagnostic performance (Figure 5G). ROC curve analysis showed that ECE1, FLT3, APPL1, RAB5C and FCGR2A all demonstrated high diagnostic performance (0.6 < AUC <0.8) (Figure 5H).

Meanwhile, the diagnostic accuracy of the model was validated using the external dataset GSE7158. The nomogram shows that the key genes have high diagnostic efficacy for the model (Figure 5D). The calibration curve indicates that the calibration dashed line is close to the model’s diagonal, suggesting good predictive value for clinical diagnosis (Figure 5E). DCA results demonstrate that these key genes have good clinical applicability (Figure 5F). ROC curve analysis shows that the model AUC value was 0.887 (95% CI: 0.732–1.000), indicating high diagnostic performance (Figure 5I). ROC curve analysis of the key genes shows that these genes have high diagnostic value (0.5 < AUC <0.7) (Figure 5J). In conclusion, this model exhibits high diagnostic performance in OP diagnosis.

### 3.5 GSEA analysis

**FIGURE 6:**
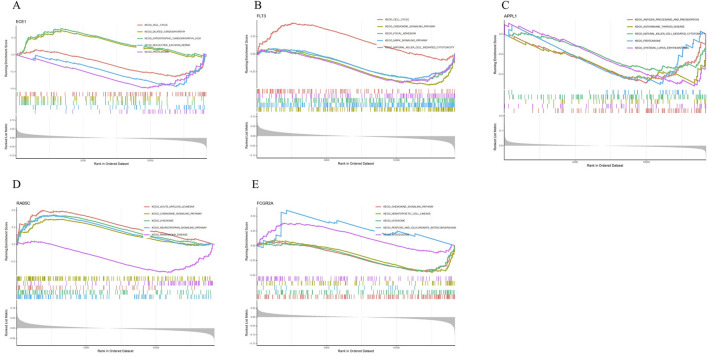
GSEA analysis. **(A)** GSEA analysis of ECE1. **(B)** GSEA analysis of FLT3. **(C)** GSEA analysis of APPL1. **(D)** GSEA analysis of RAB5C. **(E)** GSEA analysis of FCGR2A.

GSEA results show that ECE1 is mainly enriched in pathways such as Cell Cycle, Dilated Cardiomyopathy, Hypertrophic Cardiomyopathy, Nucleotide Excision Repair, and Proteasome (Figure 6A); FLT3 is mainly enriched in pathways such as Cell Cycle, Chemokine Signaling Pathway, Focal Adhesion, MAPK Signaling Pathway and Natural Killer Cell Mediated Cytotoxicity (Figure 6B); APPL1 is mainly enriched in pathways such as Antigen Processing and Presentation, Autoimmune Thyroid Disease, Natural Killer Cell Mediated Cytotoxicity, Proteasome and Systemic Lupus Erythematosus (Figure 6C); RAB5C is mainly enriched in pathways such as Acute Myeloid Leukemia, Chemokine Signaling Pathway, Lysosome, Neurotrophin Signaling Pathway and Parkinson’s Disease (Figure 6D); FCGR2A is mainly enriched in pathways such as Chemokine Signaling Pathway, Hematopoietic Cell Lineage, Lysosome, Pentose and Glucuronate Interconversions, and Spliceosome (Figure 6E).

### 3.6 Immune cell infiltration analysis

**FIGURE 7:**
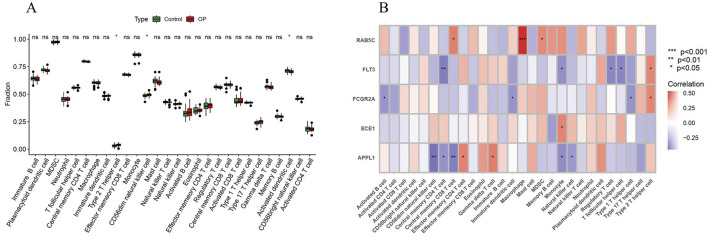
Immune cell infiltration analysis. **(A)** Boxplot analysis of 28 immune cell infiltrations between the OP group and the control group. **(B)** Heatmap of the correlation analysis between key genes and immune cells. *P < 0.05; ns denotes no significance.

The boxplot results show that, compared with the control group, Type 2 T helper cells and CD56dim natural killer cells are significantly upregulated in the OP group, while activated dendritic cells are significantly downregulated (Figure 7A). Additionally, the correlation analysis between key genes and immune cell infiltration shows that RAB5C is positively correlated with Central memory CD8 T cells, Macrophages and MDSCs; FLT3 is positively correlated with Type 2 T helper cells, but negatively correlated with Central memory CD4 T cells, Monocytes, Regulatory T cells and T follicular helper cells; FCGR2A is positively correlated with Central memory CD8 T cells, but negatively correlated with Immature dendritic cells and Type 1 T helper cells; ECE1 is positively correlated with Monocytes; APPL1 is positively correlated with Effector memory CD4 T cells and Gamma delta T cells, but negatively correlated with CD56dim natural killer cells, Central memory CD4 T cells, Central memory CD8 T cells, Monocytes and Natural killer cells (Figure 7B).

### 3.7 Construction of the drug regulation network and ceRNA network

**FIGURE 8:**
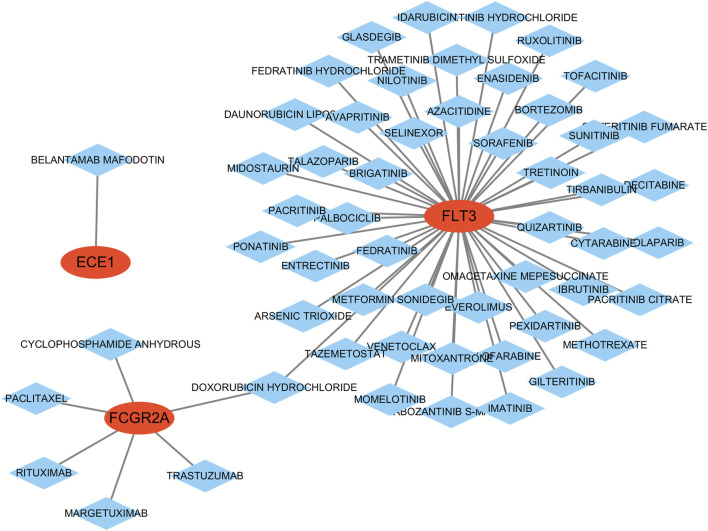
Construction of the drug-gene interaction network. The red ovals represent key genes, and the blue diamond represent predicted drugs.

The drug-gene interaction analysis results show that one drug targets ECE1; 49 drugs target FLT3; six drugs target FCGR2A; no potential drugs were found for APPL1 and RAB5C (Supplementary Table S5). Additionally, the data was visualized using Cytoscape (Figure 8).

**FIGURE 9:**
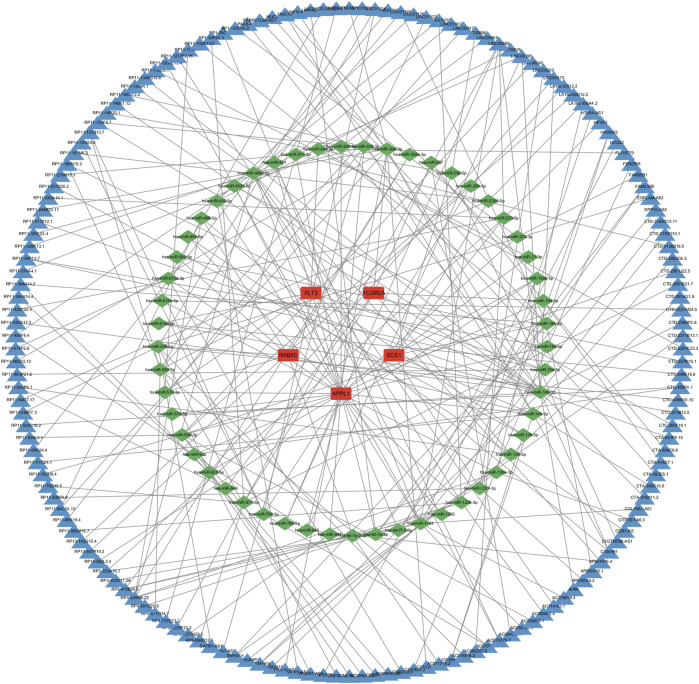
Construction of the ceRNA network. The red rectangles represent key genes, the green diamonds represent miRNAs, and the blue diamond represent lncRNA.

To further explore the potential regulatory mechanisms of key genes, a lncRNA-miRNA-mRNA regulatory network was constructed. In this study, the miRNA targets of the five key genes were predicted using three databases: miRanda, miRDB and TargetScan. Subsequently, the lncRNA targets were predicted using the spongeScans database, and the ceRNA network was constructed using Cytoscape software (Figure 9). In conclusion, the ceRNA network constructed in this study may help further understand the potential regulatory mechanisms of OP, providing new directions for disease treatment.

## 4 Discussion

OP is a systemic bone disease in middle-aged and elderly individuals, characterized primarily by low bone mass and disruption of bone microstructure, which increases the risk of fractures (Ensrud and Crandall, 2017). Due to the early symptoms of OP and the lack of clear diagnostic indicators, patients who experience fractures often suffer from severe pain, reduced mobility, and a decline in their quality of life (Yu and Xia, 2019; Alimy et al., 2024). It is estimated that approximately nine million fractures occur annually among OP patients worldwide (Yaacobi et al., 2017). Previous studies have suggested that dysregulation of circadian rhythm-related genes may contribute to the development of OP, but the underlying molecular mechanisms remain incompletely understood (Li et al., 2016). In this study, we integrated bioinformatics and machine learning approaches for the first time to identify circadian rhythm-related biomarkers associated with OP, providing new insights into its pathogenesis.

In this study, 140 CRRDEGs were identified through bioinformatics, and GO and KEGG enrichment analyses were performed. GO enrichment analysis revealed that these genes were primarily involved in immune-related processes, including the regulation of innate immune response and regulation of T cell activation. Previous studies have demonstrated that activation of innate immune cells can promote the release of inflammatory cytokines, which in turn induce osteoclast differentiation and play a critical role in the pathogenesis of OP (Saxena et al., 2021). Moreover, T cells can influence the differentiation and activity of osteoblasts and osteoclasts through paracrine signaling, thereby affecting bone remodeling (Fischer and Haffner-Luntzer, 2022). KEGG enrichment analysis indicated that these genes were associated with the insulin signaling pathway and insulin resistance. The insulin signaling pathway has been shown to regulate the activity of osteoblasts and osteoclasts, thus influencing bone metabolism (Fulzele et al., 2010). In addition, insulin resistance has been linked to structural abnormalities in bone tissue and reduced bone mineral density (Jia et al., 2024). Interestingly, circadian rhythms play a critical role in regulating insulin sensitivity in peripheral tissues (including bone),disruption of circadian rhythms may exacerbate metabolic dysfunction and accelerate bone loss (Luo et al., 2021).Collectively, these findings suggest that circadian rhythm-related genes may contribute to the development of OP, providing a theoretical basis for early diagnosis and therapeutic.

To identify key genes closely associated with circadian rhythm in OP, we used four machine learning algorithms and compared the results, determining the top five genes from the SVM algorithm as key genes for OP: ECE1, FLT3, APPL1, RAB5C and FCGR2A. Endothelin-converting enzyme 1 (ECE1) is a highly specific metalloprotease that is abundantly expressed in endothelial cells of various organs, such as the brain, heart, liver, adrenal glands, and kidneys (Kuruppu and Smith, 2012; Xu et al., 1994) Additionally, ECE1 can cleave big endothelin-1 to produce the bioactive endothelin-1 (ET-1) (Arfian et al., 2020). ET-1 has been shown to promote osteoblast differentiation and mineralization, thereby influencing bone growth (Johnson et al., 2014). Interestingly, alterations in the activity of the ECE1 gene may affect bone density, thus contributing to the progression of OP (Hansen et al., 2019). FMS-like tyrosine kinase 3 (FLT3) is a type III receptor tyrosine kinase expressed in hematopoietic cells, involved in the regulation, maintenance, proliferation, and differentiation of cells (Liu and Gu, 2024; Negotei et al., 2023). Previous studies have indicated that FLT3 may influence bone density and the occurrence of fractures in postmenopausal women, thereby affecting the progression of OP (Koh et al., 2007). Hu et al. found that FLT3 may be involved in ferroptosis in OP, although its exact mechanism remains unclear (Long et al., 2024). Interestingly, FLT3 ligands can promote osteoclast differentiation, thus affecting bone remodeling in arthritis (Svensson et al., 2016). APPL1 is an adaptor protein that influences cellular functions, such as cell proliferation, migration, and adhesion, by regulating intracellular transport and signal transduction pathways (Wen et al., 2020; Diggins and Webb, 2017). Increasing evidence suggests that APPL1 plays a significant role in the pathogenesis of OP. Zhang et al. found that the expression of APPL1 is reduced in OP and negatively regulates adipogenic differentiation in human mesenchymal stem cells (Zhang et al., 2022). Yuan et al. confirmed the downregulation of APPL1 expression in OP and its positive regulation of osteogenic differentiation in bone marrow mesenchymal stem cells (Yuan et al., 2023). Notably, APPL1 also plays a critical regulatory role in bone metabolism. Lin et al. found that the knockout of APPL1 may affect adipogenesis and osteoblast differentiation in bone marrow mesenchymal stem cells (Lin and Dong, 2020). In this study, APPL1 may be a potential therapeutic target for OP and circadian rhythms. RAB5C is a member of the Rab protein family, primarily located on the cell membrane, and plays a crucial role in endocytosis, membrane protein recycling, and signal transduction (Koop et al., 2023; Wang et al., 2024). Additionally, RAB5C may be involved in regulating various immune and inflammatory responses (Prashar et al., 2017). Zhang et al. found that RAB5C may play an important role in the onset and progression of ankylosing spondylitis by regulating immune cell function (Zhang et al., 2021). Interestingly, RAB5C may be involved in osteoclast polarization and the regulation of bone resorption activity (Zhao et al., 2002). Furthermore, RAB5C may play a key role in chondrogenesis. Studies have shown that the knockout of RAB5C can enhance the chondrogenic potential of chondrocyte progenitor cells (Janssen et al., 2021). Notably, RAB5C may have an important role in postmenopausal OP, though its exact mechanism remains unclear (Wang et al., 2020). FCGR2A is a member of the immunoglobulin Fc receptor family, primarily located on the surface of macrophages and neutrophils, and plays a crucial role in the phagocytosis and clearance of immune complexes (Niu et al., 2024; Szpakowicz et al., 2023). Additionally, FCGR2A is associated with various autoimmune diseases, such as rheumatoid arthritis (Márquez Pete et al., 2021), systemic lupus erythematosus (Cornwell et al., 2023), and ulcerative colitis (Zhang et al., 2016). Interestingly, FCGR2A has been shown to play a key role in regulating macrophage polarization. Luo et al. found that the knockout of FCGR2A can inhibit M1 macrophage polarization and NF-κB phosphorylation, while enhancing M2 polarization (Luo et al., 2024). Notably, FCGR2A is involved in osteoclast differentiation and may influence bone metabolism (Zhu et al., 2025). Increasing evidence suggests that FCGR2A may play a significant role in the pathogenesis of OP. Xia et al. identified FCGR2A as a potential target for OP diagnosis and treatment through bioinformatics (Xia et al., 2017). Another study indicated that the transcriptional activity of FCGR2A is reduced in OP bone tissue, which may provide new insights into the OP microenvironment (Balla et al., 2009). The above studies indicate that these key CRRDEGs play a crucial role in the occurrence and development of OP, to provide novel directions for advancing the understanding of OP pathogenesis.

Dysregulation of immune cells forms an important basis for immune dysfunction and plays a pivotal role in the development of OP (De Martinis et al., 2006). In this study, we found that Type 2 T helper cell、CD56dim natural killer cell and activated dendritic cell may be closely associated with the pathogenesis of OP. Th2 cells are known to contribute to bone homeostasis by producing anti-inflammatory cytokines such as IL-4 and IL-13, thereby suppressing osteoclast differentiation and bone resorption (Srivastava et al., 2018). NK cells, as components of the innate immune system, have been linked to cellular senescence and skeletal aging (Brauning et al., 2022). Dendritic cells interact with both osteoclasts and osteoblasts, and may influence bone remodeling and skeletal homeostasis (Ding et al., 2025). Moreover, we analyzed the correlation between key circadian-related genes and immune cell infiltration. The results showed that RAB5C was associated with macrophages, FLT3 with Type 2 T helper cell, FCGR2A with central memory CD8^+^ T cells, ECE1 with monocytes, and APPL1 with CD56dim natural killer cell. These findings provide new insights into the relationship between OP and immune cell dynamics.

In addition, the drug-gene interaction analysis suggested multiple approved agents that may target the key genes identified in our model, particularly FLT3 and FCGR2A. These drugs are potentially involved in modulating osteoimmune responses and regulating bone homeostasis, thereby offering promising directions for therapeutic intervention. While these interactions are computational predictions, they provide a valuable starting point for subsequent experimental validation and clinical exploration.

This study also has some limitations. First, this study was based on publicly available datasets with relatively small sample sizes, which may affect the statistical power and generalizability of the findings. Therefore, future studies should include larger sample sizes and independent datasets to validate the accuracy and reliability of the results. Secondly, although the model was validated on an independent dataset, potential algorithmic biases may still exist due to factors such as parameter selection and data preprocessing. In future studies, we plan to adopt ensemble learning methods and perform external validation using independent cohorts to further improve the model robustness and generalizability. Finally, this study lacks experimental validation, which is essential for confirming the biological functions of the key genes in osteoporosis and circadian rhythm regulation. Therefore, we plan to conduct both *in vitro* and *in vivo* experiments in future studies to further investigate their functional mechanisms.

## 5 Conclusion

In this study, we identified five key genes related to the circadian rhythm in osteoporosis (RAB5C, ECE1, FLT3, FCGR2A, and APPL1) using bioinformatics and machine learning approaches. The diagnostic model constructed based on these five genes showed high diagnostic performance. In addition, we predicted potential regulatory mechanisms involving the interactions between the key genes and immune cells, drugs, and ceRNA networks. Our findings may provide potential targets for the early diagnosis and treatment of OP. However, more experiments and integrated multi-omics data are needed to validate these findings.

## Data Availability

The datasets presented in this study can be found in online repositories. The names of the repository/repositories and accession number(s) can be found in the article/Supplementary Material.
